# Reclaiming Agriceuticals from Sweetpotato (*Ipomoea batatas* [L.] Lam.) By-Products

**DOI:** 10.3390/foods13081180

**Published:** 2024-04-12

**Authors:** Tiange Liu, Qingtong Xie, Min Zhang, Jia Gu, Dejian Huang, Qinghe Cao

**Affiliations:** 1 National University of Singapore (Suzhou) Research Institute, 377 Linquan Street, Suzhou 215123, China; min.zhang@nusri.cn (M.Z.); jia.gu@nusri.cn (J.G.); dejian@nus.edu.sg (D.H.); 2 Department of Food Science and Technology, National University of Singapore, 2 Science Drive 2, Singapore 117542, Singapore; e0966402@u.nus.edu; 3 Xuzhou Institute of Agricultural Sciences in Jiangsu Xuhuai District, Xuzhou 221131, China; caoqinghe@jaas.ac.cn; 4 Key Laboratory of Biology and Genetic Breeding of Sweetpotato, Ministry of Agriculture and Rural Affairs, Xuzhou 221131, China

**Keywords:** sweetpotato by-products, functional properties, bioactive ingredients, extraction technology, polyphenols, dietary fibres

## Abstract

Sweetpotato (SP, *Ipomoea batatas* [L.] Lam.) is a globally significant food crop known for its high nutritional and functional values. Although the contents and compositions of bioactive constituents vary among SP varieties, sweetpotato by-products (SPBs), including aerial parts, storage root peels, and wastes generated from starch processing, are considered as excellent sources of polyphenols (e.g., chlorogenic acid, caffeoylquinic acid, and dicaffeoylquinic acid), lutein, functional carbohydrates (e.g., pectin, polysaccharides, and resin glycosides) or proteins (e.g., polyphenol oxidase, β-amylase, and sporamins). This review summarises the health benefits of these ingredients specifically derived from SPBs in vitro and/or in vivo, such as anti-obesity, anti-cancer, antioxidant, cardioprotective, and anti-diabetic, evidencing their potential to regenerate value-added bio-products in the fields of food and nutraceutical. Accordingly, conventional and novel technologies have been developed and sometimes combined for the pretreatment and extraction processes aimed at optimising the recovery efficiency of bioactive ingredients from SPBs while ensuring sustainability. However, so far, advanced extraction technologies have not been extensively applied for recovering bioactive compounds from SPBs except for SP leaves. Furthermore, the incorporation of reclaimed bioactive ingredients from SPBs into foods or other healthcare products remains limited. This review also briefly discusses current challenges faced by the SPB recycling industry while suggesting that more efforts should be made to facilitate the transition from scientific advances to commercialisation for reutilising and valorising SPBs.

## 1. Introduction

Sweetpotato (SP, *Ipomoea batatas* [L.] Lam.), as a member of the *Convolvulaceae* family, is the sixth most significant staple crop worldwide, particularly in sub-Saharan Africa, parts of Asia, and the Pacific islands. In developing nations, it occupies the largest cultivated area among root crops [1]. According to statistics of the Food and Agriculture Organization of the United Nations (FAO) in 2022, global SP cultivation reached more than 7 million hectares, yielding a total production of 86 million tonnes [2], and Asia accounted for 61% of this overall production. To date, SP has been extensively cultivated in 112 countries. The great importance of SP lies in its capacity to thrive with minimal inputs, a short maturity period, strong environmental adaptive capacity, and the potential for multiple harvests within a year [3]. Moreover, it provides higher edible energy per hectare per day compared to wheat, rice, and cassava, thus serving as an excellent source of carbohydrates and other essential nutrients [1].

The storage roots, stems, and leaves of SP are edible parts rich in nutrients (e.g., starch, dietary fibres, proteins, vitamins, and minerals) and phytochemicals (e.g., polyphenols, carotenoids, and lutein) benefiting health. Bioactive properties of SP (mainly roots and leaves) have been reported by in vitro and in vivo research and cover broadly from anti-oxidant, anti-obesity, anti-diabetic, anti-inflammatory, anti-cancer, to cardioprotective activity [4]. It is worth noting that SP has high genetic polymorphism as well as diverse phenotypical characteristics in the storage root and aerial part (e.g., flesh/skin/leaf colour, size, shape, texture, and taste), which is associated with the composition of nutrients and bioactive phytochemicals [5]. For instance, orange-fleshed SP varieties are rich in beta-carotene that scarcely exists in the white-fleshed varieties, while the purple-fleshed varieties are superior sources of anthocyanins.

Despite the high nutritional and functional values of SP, a variety of SP by-products (SPBs) are generated throughout the food supply chain, including stems, leaves, storage root peels, trimmings, damaged storage roots, and the waste of residue and water from starch production, which are determined by processing methods and desired products. Only small-scale green by-products from cultivation are utilised as vegetables or low-value animal feed. The annual global food losses and waste for roots or tubers amount to 45% according to FAO’s report in 2019 [6]. In addition to direct consumption, SP is processed mainly for starch production [7]. The total starch content accounts for 57% to 90% of dry weight (DW) [8]. SP starch has been frequently used not only as a stabiliser, water-holding agent, and thickener in the food industry to prolong the shelf-life of food products, but also as a raw ingredient for producing sweetpotato starch noodles, vermicelli, sugar syrups, alcoholic beverages, monosodium glutamate, citric acid, lactic acid, and bio-ethanol [9]. However, the manufacturing process of SP starch simultaneously leads to a large amount of waste, which is mainly SP wastewater, which contains soluble fibre, micronutrients, and proteins, and solid residue, which is rich in fibre. Around 4.5–5.0 tonnes of fresh residue by-products from SP are produced per tonne of starch manufactured [7], and approximately 6 m^3^ of water is used per tonne of SP storage roots [10]. Considering the composition and physiochemical properties of SPB, its massive disposal, unfortunately, is a serious waste of bioresource, but also causes elevated levels of chemical oxygen demand, posing potential environmental hazards [11].

**Figure 1:**
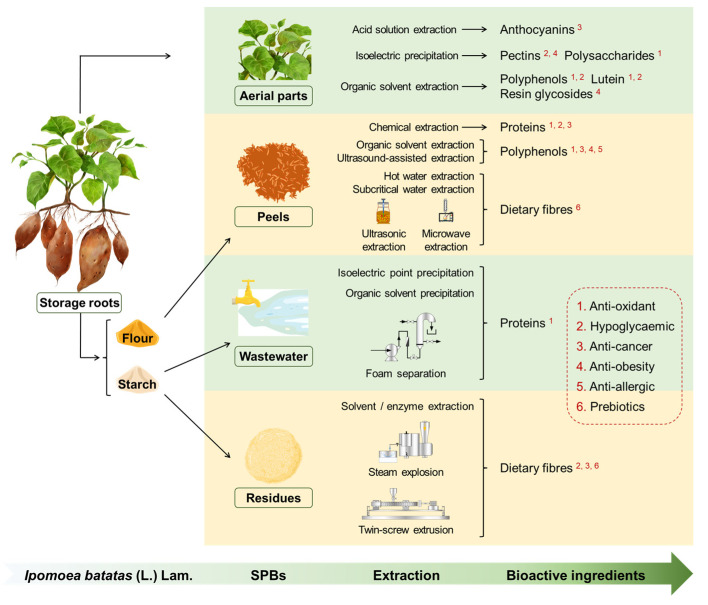
Schematic summary representing the recycling process of food and nutraceutical ingredients from sweetpotato by-products.

Accumulating research on SPBs has investigated the components extracted from SP and their biological activities. The relevant reviews summarised the chemical composition and bioactivities of SP edible parts, without the spotlight centred on cultivation and processing wastes from SP for health effects [10,12,13,14,15]. In this review, databases including Web of Science, Google Scholar, and Scopus have been used for identifying the hot spot of the recent research on SPBs. We focus on the sustainable management of SPBs, especially from the perspective of the extraction and valorisation of major health-promoting constituents from SPBs, providing some references for their application in food and nutraceutical industries (Figure 1). The research advances in other application scenarios than human health improvement are beyond the scope of this review.

## 2. SP Aerial Parts

Aerial parts of the SP can be harvested 3–4 times per year. Considering the similar annual yield of SP storage root, most of the aerial parts have been discarded, which poses a significant loss of natural resources, environmental pollution, and potentially, increases the risk of pest spread. Traditionally, the vines are used as livestock feed, and in some areas of Asian countries and the US, the young stem tip and leaves of certain types of SP are consumed as a vegetable. Table 1 shows that the aerial part of SP has good nutritional value, which may vary greatly based on the cultivars [16,17]. The moisture contents of SP leaves from the 53 cultivars summarised in Table 1 were higher than 83.28 g/100 g fresh weight. Sun et al. demonstrated that the levels of crude protein, crude fibre, crude fat, carbohydrates, and ash in the leaf samples ranged from 16.69–31.08 g/100 g DW, 9.15–14.26 g/100 g DW, 2.08–5.28 g/100 g DW, 42.03–61.36 g/100 g DW, and 7.39–14.66 g/100 g DW, respectively. Although the same measurement (AOAC method 976.05) was performed, Hong et al. reported generally higher contents of crude protein in leaves from 13 additional SP cultivars, ranging from 28.01 ± 0.19 to 38.52 ± 0.33 g/100 g DW. Also, the aerial part of SP is an excellent source of a variety of bioactive compounds, such as phenolic acids (e.g., caffeic acid (CA) derivatives, caffeoylquinic acid (CQA) derivatives, and chlorogenic acid), dietary fibre, carotenoids, flavonoids (e.g., anthocyanins, quercetin, myricetin, and fisetin), and polysaccharides [18]. Polyphenols, dietary fibre, and lutein, are the most widely studied compounds derived from SP aerial parts and thereby discussed in detail in this section, including their specific types, contents, biological activities, and extraction methods.

### 2.1. Polyphenols

#### 2.1.1. Types and Contents

**Figure 2:**
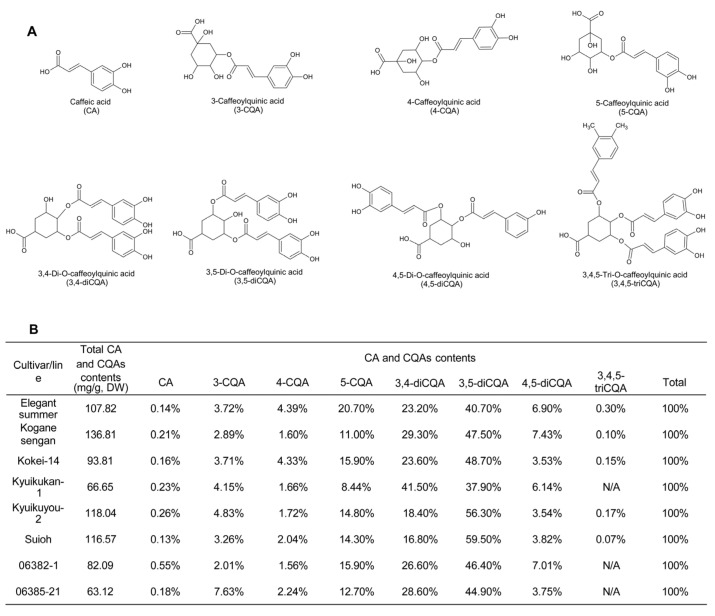
Chemical structures (**A**) of the major caffeoylquinic acid derivatives and average contents of caffeic acid (CA) and caffeoylquinic acid (CQA) (**B**) in SP leaves [26,30].

**Figure 3:**
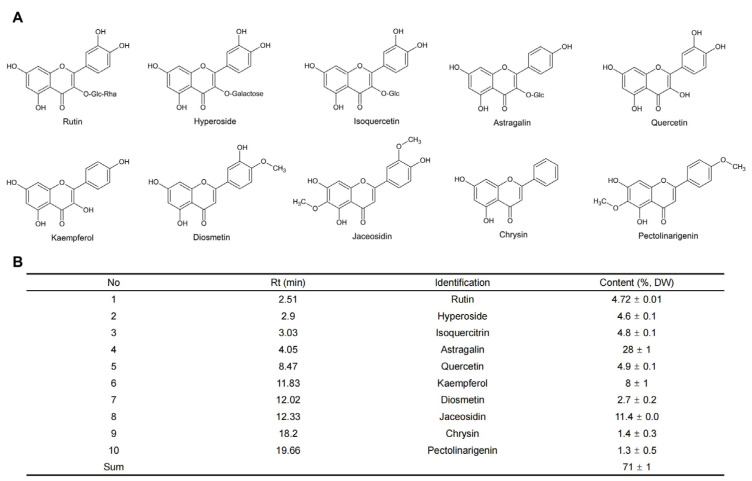
Chemical structures (**A**) and contents (**B**) of the major flavonoids by UHPLC-DAD in SP leaves [25]. Rha: Rhamnopyranosyl, Glc: Glucose.

The leaves of SP contain higher amounts of polyphenols than tubers, peels, and other vegetables [20,21]. The total polyphenol content (TPC) varies greatly among different varieties [22,23]. The major polyphenols in SP leaves include phenolic acids and flavonoids (Figure 2 and Figure 3) [24,25]. Among the phenolic acids, the most predominantly detected phenolic acids were di-, followed by mono-, and tri-O-caffeoylquinic acids or caffeic acid, with 3,5-diCQA being the most abundant by HPLC [24,26]. Figure 2 shows the major caffeic and chlorogenic acids in the eight varieties of SP leaves, with their chemical structures. Meng et al. [27] discovered 12 types of chlorogenic acid isomers from vegetable and conventional SP vine tips which could be used for distinguishing between species. Rutin, hyperoside, isoquercitrin, astragalin, quercetin, kampferol, diosmetin, jaceosidin, chrysin, and pectolinarigenin were detected as the SP leaf flavonoids, and astragalin showed the highest content by UHPLC–Orbitrap–MS/MS and UHPLC-DAD [25]. As for anthocyanins, the leaves of three SP varieties contained anthocyanin 3-(6,6’-dicaffeoyl-sophoroside)-5-glucoside and anthocyanin 3-(6,6’-caffeoyl-p-hydroxybenzoyl-sophoroside)-5-glucoside, which accounted for up to half of the total anthocyanins [28,29].

#### 2.1.2. Bioactivity of SP Leaf Polyphenols

SP leaf polyphenols have a variety of biological activities, and their antioxidant capacity has been the most widely recognised. In general, DPPH (2,2-diphenyl-1-picrylhydrazyl), ABTS (2,2′-azino-bis (3-ethylbenzothiazoline-6-sulfonic acid)), and FRAP (ferric ion reducing antioxidant potential) assays are most frequently used for evaluating the antioxidant activity. Jia et al. [31] demonstrated that TPC in SP leaves was positively correlated with antioxidant activity as measured by ABTS and FRAP, while total anthocyanin content was negatively correlated with DPPH radical scavenging activity and positively correlated with FRAP values only. Taira et al. [30] found that phenolic acids, such as CA, andCQA derivatives, in SP leaves inhibited peroxyl radical (AMVN)-induced oxidation of low-density lipoprotein (LDL) in vitro and the antioxidant activity correlated with the total amount of CQA. This result suggests that CQA derivatives from SP leaves may prevent the development of atherosclerosis. Chlorogenic acid has also been evidenced to reduce hepatic lipid deposition in a mouse model of non-alcoholic fatty liver disease via inhibiting ALKBH5 activity [32]. In human HaCaT cell lines, water extract of SP leaves attenuated hydrogen-peroxide-induced cytotoxicity. The antioxidative effects differed from SP varieties and could be contributed by TPC [33].

SP leaf polyphenols also showed hypoglycaemic potential by effectively inhibiting the activities of α-glucosidase and α-amylase in vitro, and this inhibitory effect even seemed stronger than that of the positive control acarbose [25]. Among 13 phenolic acids and 10 flavonoids identified in leaves of SP cultivar Simon No. 1, ethyl caffeate and quercetin showed the strongest inhibitory effect against both α-glucosidase and α-amylase. The inhibition mechanism may involve the binding of phenolic acids to specific amino acid residues located on the enzyme surface, leading to a conformational change and distortion of the active site, ultimately resulting in enzyme activity inhibition. The flavonoids can also interact with the active site of the enzyme, thereby inhibiting the binding between the substrate and enzyme, as well as suppressing the formation of glycosylated end products.

Additionally, the anthocyanins present in SP leaves and chlorogenic acids in SP vine tips have demonstrated anti-cancer activities. Several studies have indicated that these anthocyanins exhibit potential antiproliferative properties against MCF-7, HCT-116 and HeLa cancer cells [4]. They were also capable of inducing apoptosis in cancer cells and displayed significantly higher efficacy against colon and cervical cancer cells. Structurally, the presence of at least two hydroxyl groups on the anthocyanin B ring is crucial for its activity [34]. SP vine tip chlorogenic acid of high purity exhibited potent anti-breast-cancer properties in MB231 and MCF7 cell lines in vitro by suppressing their clonogenesis, migration and invasion [27].

Despite multiple health-promoting functions of SP leaf polyphenols, a major concern regarding their survival under physiological digestive conditions remains. The poor bioavailability greatly hinders their subsequent absorption and bioactive effects in vivo. For instance, the bioavailability of anthocyanins in human pharmacokinetic studies ranges from less than 0.005% to 1.2%, regardless of the dosage given, and is determined based on the un-metabolised parent compound [35]. During simulated gastrointestinal digestion, non-hydrolysed anthocyanins exhibited greater stability in comparison to those that underwent alkali hydrolysis [36]. Chen et al. [37] reported a 13.36% bioavailability of SP leaf polyphenols after in vitro simulated gastrointestinal digestion. The dynamic high-pressure microfluidisation (DHPM) extract of SP leaf demonstrated stable TPC during simulated gastric digestion but a significant decrease after intestinal digestion, potentially attributed to the formation of phenolic derivatives or degradation. Therefore, it is important to assess the bioactivity of SP leaf polyphenols and their concentration in blood by subjecting them to appropriate digestive and absorption models before speculating the in vivo health benefits.

#### 2.1.3. Pretreatment and Extraction Methods

Freshly harvested SP leaves have a high moisture content (85–90%) and are susceptible to water loss, decay, and surface discolouration during storage. These factors not only reduce their nutritional and bioactive value but also hinder further processing. To address this issue, drying SPBs into powder can prolong their shelf life for industrial applications. Jeng et al. [38] discovered that freeze-dried SP leaves exhibited the highest concentration of caffeoylquinic acid derivatives (147.84 mg/g) while drying at 70 °C (58.26 mg/g) and 100 °C (20.53 mg/g) significantly decreased the content of these compounds. Therefore, employing low-temperature drying (< 30 °C) may be an appropriate approach to preserve certain bioactive ingredients of SP leaves. Furthermore, the form of lyophilised powder confers easier storage and transportation of SP leaves and vines. DHPM is a reported technology that utilises a combination of high-speed impact, high-frequency vibration, instantaneous pressure drop, strong shear, and ultra-high pressure [39]. It is primarily employed for modifying macromolecules such as proteins and dietary fibres. Applying DHPM to SP leaves disrupts their cellular structure, facilitating the release and dissolution of phenolic compounds within the cells. Consequently, this process improves both the extraction efficiency and antioxidant activity.

The extraction of polyphenols from SP leaves is primarily conducted using organic solvents or acid solutions. Acid solution extraction is more commonly employed for anthocyanin extraction [28,29,31]. Available studies on the selection of organic solvents indicate that the recovery rate and antioxidant activities of SP leaf polyphenols are significantly influenced by the choice of extracting solvents [40]. An amount of 50% acetone exhibited the highest crude extract yield, the greatest total phenolic content in the extract, and the strongest DPPH scavenging activity. The main components identified are CQA and its derivatives. However, for optimal recovery of flavonoids, it was found that 70% ethanol yielded the best results. Therefore, when choosing a solvent, consideration should be given to both the main components, activities of the target extract, cost effectiveness, and environmental benefits. Such methods are needed for industrial scale extraction operation.

Due to their sensitivity to unfavourable conditions, such as temperature, light, and pH, polyphenols are prone to degradation during the processes of product extraction, processing, and storage. It has been demonstrated that the main component of SP leaf polyphenols, chlorogenic acid, undergoes hydrolysis in both alkaline and strongly acidic environments, leading to a reduction in the number of caffeoyl groups and subsequently diminishing the antioxidant activity of the extract. The antioxidant activity of SP leaf polyphenols experiences a significant decrease under high-temperature and prolonged treatment conditions (100 °C heat treatment for 90 min); however, light exposure and low-temperature heat treatment (50 °C and 65 °C) exert little impact on their antioxidant activity [41].

### 2.2. Functional Carbohydrates and Their Derivatives

Natural endogenous carbohydrates in foods cannot only serve as an energy supplier, but also, importantly, bring multiple health benefits by enhancement of the immune system; reduction of blood lipid levels; regulation of cellular signalling (communication), cell malignancy, and host-pathogen interactions; and reshaping human gut microbiota, etc., which are defined as “functional carbohydrates” [42,43]. The types of functional carbohydrates, to date, mainly include dietary fibre, active polysaccharides and oligosaccharides, sugar alcohols, and resistant/slow-digestible starch.

#### 2.2.1. Types and Contents

In the aerial part of SP, SP leaves exhibit the highest concentration of soluble dietary fibre [14]. According to a study from Mosha et al., SP leaves might contain an average of 38% dietary fibre on a DW basis [44], while other studies have reported an average of 6% soluble dietary fibre in SP fresh leaves [45]. Pectin is the main fibre component in SP leaves, constituting approximately 1.028% (DW) and primarily composed of galacturonic acid, arabinose, galactose, and rhamnose with a Mw value around 398.08 kDa. Notably, pectin derived from SP leaves possesses good swelling capacity and water-holding ability, making it suitable for enhancing viscosity and preserving freshness in food products [46]. Apart from pectin, polysaccharides make up the remaining dietary fibre content in SP leaves. These polysaccharides primarily consist of monosaccharide units including rhamnose, glucuronic acid, glucose, galacturonic acid, galactose, xylose, and arabinose [47]. Among these units, glucose and galactose dominate with a combined content exceeding 60%.

SP also contains multiple homologues of resin glycosides. Toy et al. [48] found the proposed structure of the resinous glycosides in SP leaves was either a pentasaccharide or tetrasaccharide, comprising one unit each of fucose/glucose and three or four units of rhamnose. The fucose/glucose and rhamnose units were connected through type A or type B linkages, forming a cyclic lactone ring. The fatty acid side chains in the structure of resin glycosides range from 4 to 12 carbons and could be short-chain aliphatic acid, arylalkyl acid, or saturated fatty acid side chains.

#### 2.2.2. Bioactivities

Studies have demonstrated that the pectin present in SP leaves exhibited superior bile absorption and emulsification properties in vitro compared to commercially available oat beta-glucan, thereby facilitating interactions between bile salts and fats while reducing cholesterol levels [46]. Additionally, the incorporation of reclaimed SP leaf pectin decreased the starch digestibility in vitro of wheat flour and rice noodles in a dose-dependent manner and diminished the starch hydrolysis rate by amylase. Therefore, pectin from SP leaves has the potential for developing low-glycemic-index foods.

Resin glycosides in SP leaves exhibited lipase inhibition activities in vitro, the methanolic fraction of SP leaf extract after liquid–liquid fractionation showed an IC_50_ of 39.23 ± 2.00 µg/mL [48]. The mechanism of inhibition was purely non-competitive, indicating that the resin glycosides do not compete with the substrate by binding to the active site of pancreatic lipase, but rather bind to a separate site on the free lipase or the enzyme–substrate complex. Meanwhile, in vitro fat digestion experiments confirmed that the inhibitory ability of resin glycosides on fat hydrolysis was dose-dependent, but also required sufficient time of enzyme binding for effective inhibition of lipase. Interestingly, the oral administration of a high-fat diet incorporated with 6% resin glycoside crude extract (SP storage root source) in C57BL/6J mice could effectively inhibit body weight gain, improve insulin sensitivity, reduce fat accumulation, and attenuate hepatic steatosis, but its mechanism of action seems more complicated than lipase inhibition and the interaction of resin glycosides with gut microbiota requires further investigation [49].

#### 2.2.3. Extraction Methods

Interestingly, the physicochemical and functional characteristics of polysaccharides can decide, at least partly, the strategy of pre-treatment and extraction, which in turn in-fluences their biological activities and practical applications. For example, before extracting polysaccharides from SP leaves, ultrasound and complex enzyme treatments can promote cell fragmentation and thus improve polysaccharide extraction rate, as well as produce more small-molecule polysaccharides, resulting in improved hypoglycaemic activity due to favourable binding sites for α-amylase and α-glucosidase [47]. Pectin and polysaccharides in SP leaves were mainly extracted through isoelectric precipitation [46,47], while resin glycosides were extracted using organic solvents [48,49].

### 2.3. Lutein

Carotenoids play a crucial role as pigments in the photosynthetic process of various organisms, being present in different parts of plants including leaves, roots, flowers, and fruits [50,51]. In leafy crucifers, SP leaves are a superior source of lutein (β, ε-Carotene-3, 3′ diol) which is a naturally occurring derivative of hydrocarbon carotenoids [52]. The content of lutein varies between SP varieties, types, and growth stages. For instance, on average, SP leaves contain 24.67 mg of lutein per 100 g of SP dry leaves, but only 2.70 mg in SP stems [53]. The lutein content in fresh, dried, and frozen leaf samples is 442.3, 14.7, and 179.5 mg/g, respectively [54].

The primary physiological activity of lutein lies in its antioxidant and light-filtering functions, which inhibit the formation of free radicals by quenching singlet oxygen and reduce oxidative damage through the presence of large quantities of lutein outside retinal cells. Additionally, lutein is concentrated in the macula where it absorbs and attenuates high-energy blue light to protect against light-induced damage [55]. Furthermore, studies have suggested that higher dietary intake of lutein may confer beneficial effects on cardiovascular disease risk reduction [56]. Despite extensive research on lutein’s properties, there remains limited literature regarding SP leaf-derived sources. For instance, Ahmad Safiyyu’d-din Bin Hisamuddin et al. [57] reported that luteolin from SP leaves was able to restore the ferric-reducing ability of plasma, glutathione, and total antioxidant capacity levels in the serum and retina of streptozotocin-induced diabetic rats and at 400 mg/kg, was able to restore the retina, lens, and even kidney, liver, and pancreas structures of the rats to be close to similar to those of non-diabetic rats. In addition, lutein in SP leaves showed no apparent signs of toxicity in both acute and subacute studies in mice, and no abnormalities were found in the histomorphology of the retina, kidney, liver, pancreas, and heart [58]. This suggests that lutein may be used as a functional food or even an antidiabetic agent in the future, but more studies are still needed to fully reveal the mechanisms of action.

Currently, the commercial source of lutein is the flower of the genus *Tagetes* [59], instead of SP leaves. Therefore, we have not found the published methods of its extraction in the food industry. Under laboratory conditions, the main extraction method for lutein is 80% ethanol soaking and subsequent storage in a mixture with maltodextrin after drying [57,58].

## 3. SP Storage Root Peels

The production of SP storage root flour mainly includes six stages, namely raw SP storage root selection, trimming, washing, slicing, dehydration, and milling [60]. Due to the robust structure of SP peels, which is resistant to breakage, they are commonly removed during the processing of SP flour. This step enables a shorter processing procedure and yields SP flour with enhanced processing characteristics compared to direct grinding of unpeeled storage root. However, Shaari et al. [61] demonstrated that unpeeled storage roots of Anggun 1 SP cultivar are superior materials for flour production due to higher contents of dry matter, fibre, carbohydrates, and amylose as compared to peeled storage roots. Furthermore, this peeling practice wastes an abundance of SP peels, which unfortunately, at present, have little commercial value. Increasing research evidenced the existence of dietary fibre, functional proteins, and polyphenols in SP root peels, as well as their health-promoting properties that render the SP peel suitable for incorporation as novel functional foods too [62].

### 3.1. Polyphenols

The peels of SP roots exhibit nearly three times the antioxidant activity compared to the rest of the storage root, despite already having 3.2 times more antioxidant activity than blueberries [63,64]. The major polyphenol types contained in SP peels (variety unknown) are chlorogenic acid (112.24 mg/100 g DW), eryptochlorogenic (46.28 mg/100 g DW), 3,5-dicaffeoylquinic acid (36.02 mg/100 g DW), 4,5-dicaffeoylquinic acid (28.01 mg/100 g DW), and vanillic acid (22.68 mg/100 g DW) [65].

Polyphenols from SP root peels are reported as multifunctional active substances. Firstly, they were able to scavenge DPPH and ABTS free radicals, and the scavenging efficiency of DPPH was higher than that of ABTS, which may be attributed to the higher antioxidant activity of lipophilic or chelated compounds in the polyphenols of SP peels as compared with hydrophilic compounds [66]. Also, the anti-allergic effects of SP peel polyphenols were concluded by suppressing β-Hexosaminidase release of RBL-2H3 cells [67]. These polyphenols came from Naruto Kintoki, a well-known Japanese SP variety. Phomkaivon et al. found that cooking, including baking, microwaving, and boiling, could increase the content of ombuin and 3, 5-dihydroxy-7, 4′-dimethoxyflavone with improved anti-allergic activity. Moreover, octadecylcoumarate, 7-hydroxycoumarin, and 6-methoxy-7-hydroxycoumarin isolated from SP peels showed inhibitory effects on the growth of multiple cancer cell lines, such as lung, breast, head/neck, and colon cancer cells [68]. In addition, Althwab et al. [65] developed a fermented milk with the addition of polyphenols extracted from SP peels which could reduce the levels of total cholesterol, LDL, protein, and triglycerides in the blood of hypercholesterolaemic rats. To sum up, these findings support the potential of polyphenols extracted from SP peels to be recycled as active ingredients for the development of functional products in food, pharmaceutical, neutraceutical, or cosmetic industries.

Currently, the primary method for extracting polyphenols in SP peels is based on the utilisation of organic solvents, such as ethanol, by maceration [65,67,68]. This extraction approach only considers the effects of solvent types, solvent-to-solid ratio (positive), and peel cutting depth (negative) on the extraction efficiency [63]. To mitigate the environmental impact of organic solvents, water extraction conditions were also investigated. The optimal conditions for water extraction were determined to be a solvent-to-solid ratio of 60 mL/g (solid), a temperature of 75 °C, and an extraction time of 30 min [66]. Additionally, ultrasound can be employed as an auxiliary technique for chlorogenic acid extraction from SP peels [69]. The power density of ultrasound affects the content of specific chlorogenic acid isomers. Power densities ranging from 35 to 50 W/L promote acyl migration between different caffeoylquinic acid isomers and enable obtaining extracts enriched in specific chlorogenic acids like 3-caffeoylquinic acid and 3-caffeoyl-4-feruloylquinic acid at high power densities. Therefore, this method can be utilised for extracting specific chlorogenic acids with desired activities or properties.

### 3.2. Dietary Fibres

Approximately 80% of SP peels (DW) is carbohydrate [18]. The contents of dietary fibre are 58.4% and 31.0% in the initial peels and blanched peels (DW), respectively [18]. Insoluble fibre accounts for about 50% of the total dietary fibre [70].

Cao et al. [71] showed the prebiotic role of dietary fibre from SP peels in simulated fermentation in vitro with human faecal bacteria. The addition of dietary fibre from SP peels promoted the abundance of *Bifidobacterium*, *Faecalibacterium*, and *Prevotella* and reduced the abundance of *Proteobacteria*, *Romboutsia*, and *Dorea*. Meanwhile, more short-chain fatty acids were produced, and intestinal pH was reduced. Researchers have attempted hot water extraction, microwave extraction, ultrasonic extraction, and subcritical water extraction to obtain dietary fibre from SP peels. Among these methods, subcritical water extraction yielded the highest recovery of total dietary fibre and soluble dietary fibre, while hot water extraction had the lowest recovery. However, ultrasonic extraction exhibited superior effects on improving intestinal microecological health. For instance, the dietary fibre extracted by ultrasonic-assisted method displayed the most significant effect on reducing the sugar/total sugar ratio (%) and the highest degradation rate after 24 h intestinal fermentation in vitro. Considering the negligible disparity in the recovery efficiency of dietary fibre between ultrasonic and subcritical water extraction methods, opting for ultrasonic extraction on an industrial scale may ensure enhanced functionality of dietary fibre derived from SP peels.

### 3.3. Proteins

Maloney et al. [18] compared the extracted proteins from primary peeling which was performed at the very beginning before any processing of orange-fleshed SP cultivars, and the secondary peeling after SP root blanching (Table 1). The dominant storage protein in SP peels, sporamin, showed inhibitory activities against trypsin, even after incubation with pepsin, and resistance to multiple digestive enzymes, including pepsin, trypsin, and chymotrypsin. Heat and simulated gastric digestion effectively abolished the amylase activity present in the peel. The proteins could be extracted from SP peels, including dissolving proteins by mixing blanched peelings with NaCl and subsequently precipitating with CaCl_2_ [18,72]. It is worth mentioning that both the salt solution concentration and the extraction temperature would have certain effects on the recovery of proteins. Considering that sporamin is also the primary protein reclaimed from the SPB of starch processing, its functionality is discussed in Section 4.1.2.

## 4. SPBs from Starch Processing

Apart from direct consumption and use as feed, the main use of SP is starch processing, which accounts for almost half of SP production. Although starch is the most abundant nutrient in SP storage roots (the starch content varies among different SP varieties) [73,74], a significant amount of SP solid residue is inevitable during the starch manufacturing process. Meanwhile, the commonly used water-to-material ratio is approximately 1:10 (*w*/*v*), resulting in millions of tonnes of wastewater with high organic matter content. Even with optimised measures to minimise wastewater output during starch production, its generation remains unavoidable [75]. Regrettably, proteins, carbohydrates, and other nutrients are discarded in SPB, causing a severe loss of bioresource and potential environmental pollution. Therefore, recovery and valorisation of bioactive components and nutrients from these wastes of starch production would increase economic profits and environmental friendliness for the sustainable development of the SP industry.

### 4.1. Proteins

Regardless of the differences between varieties, SP protein is the main and versatile ingredient in SPBs from starch processing, such as polyphenol oxidase, β-amylase, and storage proteins (sporamins) [76]. According to Mu et al. [77], the crude protein contained in SP ranges from 0.49% to 2.24% on a fresh weight basis, while wastewater contains approximately 1.5% crude protein. SP protein also has a well-balanced amino acid composition, with essential amino acids accounting for about 40% of the total amino acid content [77]. The amino acid score indicated that lysine was the limiting amino acid in SP proteins [78].

#### 4.1.1. Types and Contents

The proteins extracted from the wastewater were characterised by Yang et al. [79]. A total of 25 proteins were identified, with β-amylase, sporamins (sporamins A, sporamins B, and their precursors), and polyphenol oxidase (PPO) being the predominant components. These three types of SP proteins are also the primary targets for recovery in wastewater treatment processes [80,81].

#### 4.1.2. Functionality as Food Ingredients

Sporamins present as the primary storage proteins in SP roots, accounting for 60–80% of the total soluble protein content [82]. Sporamins show antioxidant properties, such as dehydroascorbate reductase and monodehydroascorbate reductase activities [83]. The structure of three sporamin proteins consists of seven common peptides and at least one unique peptide fragment, in which cysteine residue is of great importance in antiradical activities [79,84]. The potential mechanism could involve the reduction of DPPH radical and inhibition of its oxidation by sulfydryl present in SP sporamin [85]. Whether the resistance of SP sporamin to protease digestion adversely affects human nutrition remains unclear [86]. The study conducted by Zhang et al. [87] revealed that autoclaving, steaming, microwaving, boiling, and ultrasonication (in descending order of effectiveness) significantly enhanced the antioxidant peptide content of SP protein hydrolysates produced by alcalase and protease enzymes. These peptides were identified as sporamin A, its precursor, and sporamin B. Therefore, appropriate pretreatment and enzymatic hydrolysis may assist the generation of high-quality SP protein products as food ingredients.

β-amylase is one of the widely utilised enzymes in the fermentation and food manufacturing industries [79]. It constitutes approximately 5% of the total soluble proteins in SP roots, and its content and activity have been demonstrated to be positively correlated with the maturity of roots and vary between different varieties [88]. PPO plays multiple roles in phenol polymerisation, degradation, and other substances. Apart from its industrial applications, it can be employed in tea production within the food sector to enhance the flavour and quality of black tea [80]. Studies have demonstrated that PPO derived from SP sources can be used for isolating theaflavins or preparing black tea with high theaflavin content [81]. In summary, the biological activities of β-amylase and PPO have more directly contributed to food processing than human health.

#### 4.1.3. Extraction Methods

Similar difficulties as discussed in Section 2.1.3 are met when reprocessing SP wastewater and residue from starch production. Yang et al. [79] found that converting spray-drying SP starch industrial wastewater into powder enables efficient extraction of bioactive compounds. The primary techniques utilised for protein extraction and separation from wastewater generated by SP starch manufacturing include isoelectric point precipitation and organic solvent precipitation. PPO can be precipitated and separated from β-amylase within the pH range of 3.5 to 4, but both PPO and β-amylase become inactivated when the pH drops below 3. Sporamins can be separated through precipitation using organic solvents [81]. It has been demonstrated that 50% ethanol and acetone are effective in separating sporamins from PPO or β-amylase [80,81].

If the enzymes and sporamins are not the desired products but the crude protein from the wastewater, the foam separation method can be employed. Foam separation, also known as adsorption bubble separation, can concentrate surface-active substances utilising foam bubbles. This technique solely requires air or an inert gas to enhance the concentration of active substances in dilute solutions. This method for protein recovery could be influenced by various factors including protein concentration, pH level, water intake, foaming time, air flow rate, and the angle of the slanting column of foam. Mu et al. [89] demonstrated that at an initial protein concentration of 4.51 mg/mL, pH 4, water intake of 500 mL, foaming time of 100 min, air flow rate of 0.15 mg/mL, and a slanting column angle of 30°, maximum SP protein recovery and enrichment were achieved at levels of 84.1% and 1.3%, respectively. In contrast to this study, Hu et al. [11] improved foam stability by incorporating hydrophobic silica nanoparticles into the system as a control agent for facilitating proteins’ separation in low concentrations or with poor surface activity when using the foam separation method.

### 4.2. Diet Fibres

The composition of SP residue is influenced by the variety of SP, the process of starch extraction, and the level of technical expertise. However, its primary constituents remain starch, dietary fibre, and a small amount of protein [19,90]. Consequently, SP residue can serve as an excellent source of dietary fibre.

#### 4.2.1. Types and Contents

Mei et al. [19] conducted a comparative analysis on the composition of crude dietary fibre extracted from 10 different varieties of SP residue, primarily consisting of cellulose, lignin, pectin, and hemicellulose ranked in descending order of the mean content (Table 2). The authors also determined the monosaccharide composition of the extracted dietary fibre and identified eight different monosaccharides: rhamnose, arabinose, galactose, glucose, xylose, mannose, galacturonic acid, and glucuronic acid (Table 2). Glucose (46.12–66.25%) was found to be the most abundant component as a major constituent of cellulose and hemicellulose; meanwhile, glucuronic acid (14.60–34.96%) ranked second as a major constituent of pectin.

#### 4.2.2. Bioactivities

Dietary fibre is widely recognised for its biological activity as a prebiotic that significantly increases the concentration of intestinal *Bifidobacteria* and *Lactobacilli* [90,91]. More biological activities have been discovered in recent years. For example, Liu et al. [92] modified dietary fibre extracted from SP residues using alkaline hydrogen peroxide (AHP) and demonstrated its ability to inhibit α-amylase activity and consequently to reduce glucose release efficiency. Moreover, it exhibits significant inhibition of glucose diffusion rate, possibly due to its increased viscosity compared to unmodified dietary fibre (O-SPDF), forming a physical barrier that hinders glucose retention. Notably, SP dietary fibre has also shown protective effects against lead-induced kidney injury. It effectively reduces Pb concentration in both blood and kidneys of mice while preventing Pb-induced apoptosis through regulation of the AMPK/SIRT1/PGC1α pathway in mouse kidneys. These findings suggest that SP dietary fibre can serve as a natural agent for eliminating Pb or be incorporated into functional foods to prevent Pb accumulation for improved kidney health [93]. Another example is the pectin extracted from SP residue by Zhang et al. [94], which was found to possess in vitro anti-cancer properties by inhibiting the proliferation of HT-29 and Bca-37 in a dose-dependent manner.

#### 4.2.3. Extraction Methods

Dietary fibre, especially pectin, is commonly extracted by treating SP residue with acid or enzymes followed by ethanol precipitation [19,93,94,95,96]. This process effectively removes other substances such as starch and protein that may be present in the SP residue. According to Zhang et al.’s optimised pectic acid extraction method using response surface methodology, the optimal conditions for extraction included a temperature of 93 °C, an extraction time of 2.2 h, a solution pH of 1.7, and a liquid–solid ratio (*v*/*w*) of 30:1, which combined high reaction temperature, large liquid–solid ratio, and strong acidity in the solution [94]. By contrast, another study demonstrated that disodium phosphate solution can also be utilised for extracting pectin from SP residue under optimal conditions including a liquid–solid ratio of 20:1, an extraction time of 3.3 h, an extraction temperature of 66 °C, and a solution pH of 7.9 [97]. Although this method requires a slightly longer extraction time compared to the mentioned research, it involves a lower liquid–solid ratio and extraction temperature along with a weak alkalinity solution, which makes it less environmentally hazardous.

In contrast to the traditional methods of extraction, several reported technologies may increase the extraction rate of dietary fibre and change its properties, such as steam explosion. In this process, the raw material is subjected to high temperature and pressure steam for a few minutes, followed by sudden depressurisation [95]. This treatment effectively disrupts the cell walls, facilitating the release of dietary fibre from the cells. Compared to untreated methods, this approach results in an 18.78% increase in dietary fibre content. Additionally, it enhances the water absorption capacity, oil retention capacity, and swelling ability of dietary fibre due to its loose and porous structure resulting from steam explosion treatment. Another technology of dietary fibre extraction from SP residue is twin-screw extrusion, which could also lead to a significant increase from 9.63% to 29.25% in the yield of dietary fibre [98]. Specific processing conditions were applied, including a feed moisture setting of 40%, an extrusion temperature of 150 °C, a screw speed of 180 rpm, and a feed rate of 17 Hz. Simultaneously, this method could modify dietary fibre for improved absorption capacity for lipophilic components such as cholesterol and bile salts and strengthened antioxidant activity. These merits make the dietary fibre product by twin-screw extrusion highly suitable as a functional ingredient in the food industry. Additionally, the cellulose recovered from SP residue could be hydrolysed using sulfuric acid to produce cellulose nanocrystals, which exhibited an increased crystallinity of 72.53% [96]. This process effectively enhanced the adsorption capacity of cellulose for heavy metals, allowing it to be potentially applied in the areas of pharmaceutical and food additives, bio-nanocomposites, packaging, etc.

## 5. Applications

As previously mentioned, SP waste contains various bioactive substances that can be extracted and then potentially incorporated into foods or other health products. Here are some examples of edible-plant-derived bioactive compounds. Sęczyk et al. [99] demonstrated that the addition of chlorogenic acid (derived from raw coffee beans) to soya bean milk effectively enhanced its antioxidant capacity and promoted the hydrolysis of soya bean proteins by digestive enzymes, thereby improving its nutritional quality. Similarly, incorporating anthocyanins (derived from black rice) into bread inhibited starch hydrolysis, thus regulating starch digestibility, reducing starch pasting, and increasing starch crystallinity, which eventually led to changes in the microstructure of bread [100]. These active compounds have shown positive effects on food processing and nutritional value; however, their raw materials are comparatively expensive. Substituting these active ingredients with those derived from SPB would likely reduce costs to some extent and facilitate bioresource reutilisation. Li et al. [81] utilised recovered polyphenol oxidase from SP wastewater to synthesise theaflavins based on catechins from tea residues through an environmentally friendly fed-batch feeding method. At present, SP-derived products, including the whole powder [8], resistant starch [101], and puree [12], have been well utilised in industrial food manufacturing for the alteration of texture and appearance and the enhancement of nutritional properties. In contrast, the industrial application of active ingredients derived from SPBs has been still rarely reported.

## 6. Challenges and Prospects

SPB recycling still faces several challenges. First, the creation of an efficient and sustainable recycling system is critical. Secondary pollution in the recycling of SPBs should be avoided by adopting environmentally friendly technologies for SPB management. A few emerging technologies have been developed for reprocessing edible plants and medical herbs, such as flash extraction, supercritical fluid extraction, ultrasound-assisted extraction, and microwave-assisted extraction, etc. with the advantages of less energy consumption, minimised use of organic solvents, and shortened operational durations [102,103,104,105]. To date, except for SP leaves, many of these advanced technologies have not been applied specifically in reprocessing SPBs [106,107,108], which can be further explored. It is noteworthy that there is not a perfect extraction technology that would simultaneously meet both requirements from the food sector, including a shorter process and maximum yield. Excitingly, the proper combination of different extraction techniques may generate even improved results than a single successful method [109,110,111]. Second, there are limited technologies available for systematically or sequentially recovering, purifying, and concentrating multiple types of valuable compounds from SPBs or other agro-industrial waste. So far, most recycling studies primarily focus on a single component from SPBs. Likewise, the solution, but also the challenge, lies in consolidating these extraction technologies in an industrial environment in a rational and green process that maximises the recovery of active substances with high quality and purity from SPBs [112]. Moreover, considering that specific precious substances present in SPBs are susceptible to temperature fluctuations, pH variations, and other environmental conditions that may lead to diminished activity or decomposition [41], in addition to extraction, it is imperative to include the initial disposal, storage, and pre-treatment of SPBs, as well as the further conversion of isolated substances to value-added products in the entire recycling strategy for desired health-promoting effects. Finally, more efforts are needed to promote the transition from academic laboratories to industrial plants. One of the major obstacles faced by the bioprocessing industry is the lack of operational data on technological advancements for scaling up. The recovery of active substances in the laboratory is typically conducted on a small scale. When scaling up the regeneration process, its economic, environmental, and social impacts would be magnified. For instance, although the cost of SPB raw materials is negligible, the manpower, technical capability, and fund input are necessary from recollection to their management and valorisation. Importantly, the transformation and commercialisation of biowaste into bio-products will benefit greatly from the support and leadership of the national policies, as well as in combination with proper business models to meet the market demand.

## 7. Conclusions

With the increasing demand for food globally, substantial agro-industrial wastes are generated. Reclaiming agriceuticals to develop high-value-added products facilitates the reduction of food wastage. This review has summarised and discussed the research advances of SPB conversion in recent decades from the perspective of its functional values. SPBs, in the forms of SP stems, leaves, storage root peels, as well as wastewater and residues from starch processing, contain various bioactive ingredients, e.g., polyphenols, proteins, dietary fibres, etc., the potentials of which have been evidenced to be applied in food, nutraceutical, and pharmaceutical industries. Continual efforts should be made to establish a sustainable and complete recycling system of SPBs starting from SP harvesting to the generation of high-value-added bio-products, achieving improved cost-benefit advantages and simultaneously, zero waste.

## Figures and Tables

**Table 1:** The nutrient contents in sweetpotato aerial parts, storage root peels, and residues from starch production *.

Recyclable Part	Cultivar	Moisture	Crude Fibre	Crude Protein	Crude Fat	Ash	Dietary Fibre	Carbohydrate	Refs.
Aerial part	Ximeng No. 1	88.70 ± 1.81	12.76 ± 0.05	25.66 ± 0.63	3.06 ± 0.15	-	-	-	[16]
	Jinyu No. 1	88.10 ± 2.03	11.28 ± 0.02	27.53 ± 0.33	3.43 ± 0.06	-	-	-	
	Jishu	87.60 ± 0.23	11.26 ± 0.06	29.27 ± 0.02	3.99 ± 0.11	-	-	-	
	Shi No. 5	87.95 ± 1.85	11.06 ± 0.07	31.08 ± 0.09	5.13 ± 0.09	-	-	-	
	Xushu No. 55-2	87.85 ± 0.12	10.62 ± 0.05	29.08 ± 0.35	4.88 ± 0.12	-	-	-	
	Jishu No. 22	87.57 ± 0.58	12.98 ± 0.07	27.15 ± 0.13	4.90 ± 0.04	-	-	-	
	Yanshu No. 25	87.33 ± 0.93	11.26 ± 0.05	23.46 ± 0.21	4.08 ± 0.06	-	-	-	
	Xushu No. 23	84.54 ± 0.66	11.36 ± 0.00	30.53 ± 0.32	4.95 ± 0.06	-	-	-	
	Sushu No. 14	87.63 ± 0.16	11.03 ± 0.10	26.75 ± 0.16	4.47 ± 0.15	-	-	-	
	Wanshu No. 5	86.79 ± 0.19	12.45 ± 0.17	27.20 ± 0.12	5.23 ± 0.18	-	-	-	
	Longshu No. 9	86.25 ± 0.69	13.00 ± 0.02	25.71 ± 0.04	4.90 ± 0.12	-	-	-	
	Hongxinwang	87.52 ± 0.31	10.55 ± 0.54	24.72 ± 0.17	3.71 ± 0.08	-	-	-	
	Xushu No. 053601	88.92 ± 0.34	10.04 ± 0.50	23.43 ± 0.11	3.75 ± 0.01	-	-	-	
	Nongda No. 6-2	88.84 ± 1.02	9.86 ± 0.35	24.21 ± 0.17	3.84 ± 0.16	-	-	-	
	Miyuan No. 6	88.59 ± 0.53	9.25 ± 0.38	23.49 ± 0.43	3.97 ± 0.04	-	-	-	
	Yuzi No. 7	87.52 ± 0.20	10.68 ± 1.15	21.12 ± 0.25	2.24 ± 0.08	-	-	-	
	Beijing No. 553	86.75 ± 0.87	9.71 ± 1.50	22.03 ± 0.01	5.17 ± 0.10	-	-	-	
	Xinong No.1	87.78 ± 0.62	10.19 ± 0.85	18.35 ± 0.01	5.28 ± 0.15	-	-	-	
	Jishu No.04150	87.82 ± 1.16	10.24 ± 0.69	23.18 ± 0.13	4.22 ± 0.04	-	-	-	
	Pushu No.53	88.28 ± 1.02	11.33 ± 0.46	24.04 ± 0.11	4.39 ± 0.16	-	-	-	
	Xushu No. 22-1	86.81 ± 0.22	11.88 ± 0.93	22.96 ± 0.25	2.08 ± 0.06	-	-	-	
	Shangshu No. 19 (spring)	88.56 ± 0.14	10.01 ± 0.75	16.69 ± 0.09	2.94 ± 0.10	-	-	-	
	Shangshu No. 19 (summer)	87.85 ± 0.65	9.15 ± 0.49	17.92 ± 0.11	2.85 ± 0.16	-	-	-	
	Sushu No. 16	84.09 ± 0.81	12.70 ± 0.35	27.55 ± 0.35	2.37 ± 0.08	-	-	-	
	Chuanshu No. 294	87.76 ± 0.14	12.32 ± 0.74	28.57 ± 0.04	2.53 ± 0.01	-	-	-	
	Xinxiang No. 1	86.33 ± 0.90	13.11 ± 0.72	28.62 ± 0.08	2.42 ± 0.03	-	-	-	
	Xushu No. 038008	86.75 ± 3.31	11.54 ± 0.68	25.94 ± 0.06	3.17 ± 0.04	-	-	-	
	Yanzi No. 337	88.65 ± 2.56	10.33 ± 0.79	23.77 ± 0.19	3.57 ± 0.12	-	-	-	
	Shanchuanzi	88.76 ± 1.44	11.26 ± 1.19	21.46 ± 0.13	3.25 ± 0.06	-	-	-	
	Pushu No. 17	88.89 ± 1.69	14.26 ± 0.38	18.62 ± 0.11	3.16 ± 0.01	-	-	-	
	Jinong No. 2694	86.20 ± 1.44	10.82 ± 1.28	25.26 ± 0.26	3.31 ± 0.08	-	-	-	
	Fushu No. 2	88.53 ± 2.36	12.10 ± 1.02	24.59 ± 0.33	3.81 ± 0.08	-	-	-	
	Ningzi No. 23-1	88.45 ± 2.19	13.00 ± 1.02	22.76 ± 0.35	3.54 ± 0.01	-	-	-	
	Langshu No. 7-12	88.42 ± 1.90	12.40 ± 0.58	22.25 ± 0.01	3.89 ± 0.02	-	-	-	
	Jingshu No. 6	87.24 ± 2.64	12.70 ± 0.49	23.76 ± 0.07	3.27 ± 0.06	-	-	-	
	Ningzi No. 1	87.53 ± 2.55	13.59 ± 1.00	22.45 ± 0.26	3.37 ± 0.07	-	-	-	
	Yuzi No. 263	87.93 ± 0.37	13.13 ± 0.67	22.76 ± 0.01	3.22 ± 0.02	-	-	-	
	Xushu No. 26	88.15 ± 2.14	12.20 ± 1.80	22.63 ± 0.07	2.93 ± 0.16	-	-	-	
	Jishu No. 65	87.58 ± 1.53	11.81 ± 1.29	21.80 ± 0.56	3.30 ± 0.00	-	-	-	
	Xushu No. 22 (spring)	87.68 ± 1.39	12.62 ± 0.23	17.53 ± 0.29	3.04 ± 0.01	-	-	-	
	Guang2	89.67 ± 0.87	10.92 ± 0.07	33.64 ± 0.83	3.87 ± 0.64	15.62 ± 0.05	37.28 ± 0.1	36.31 ± 0.49	[17]
	Guang5	88.67 ± 1.34	9.26 ± 0.03	31.41 ± 0.69	2.75 ± 0.41	14.86 ± 0.05	38.87 ± 0.33	41.98 ± 0.55	
	Ecai1	87.92 ± 0.43	9.82 ± 0.08	35.66 ± 0.2	4.28 ± 0.92	13.43 ± 0.15	40.32 ± 0.1	36.79 ± 1.24	
	Ecai10	89.95 ± 0.16	10.63 ± 0.01	38.52 ± 0.33	4.25 ± 0.33	16.61 ± 0.12	38.71 ± 0.01	30.13 ± 0.74	
	Zhecai1	89.89 ± 0.36	9.74 ± 0.12	35.45 ± 0.31	2.78 ± 0.23	15.51 ± 0.03	38.48 ± 0.42	36.75 ± 0.88	
	Zhe726	90.01 ± 1.2	9.91 ± 0.09	33.65 ± 0.34	2.74 ± 0.22	14.61 ± 0.18	39.06 ± 0.3	38.15 ± 0.3	
	Fu18	88.41 ± 0.98	10.19 ± 0.02	36.44 ± 0.25	2.78 ± 0.23	16.48 ± 0.03	38.91 ± 0.04	34.01 ± 0.19	
	Fu22	87.37 ± 0.82	10.11 ± 0.02	28.01 ± 0.19	2.74 ± 0.22	16.47 ± 0.01	41.45 ± 0.11	42.64 ± 0.12	
	Fu23	87.96 ± 2.03	11.4 ± 0.06	36.16 ± 0	2.75 ± 0.06	15.45 ± 0.07	40.35 ± 0.14	34.22 ± 0.19	
	Taninong71	88.24 ± 0.13	10.2 ± 0.07	35.49 ± 0.07	3.3 ± 0.21	15.93 ± 0.07	40.06 ± 0.13	35 ± 0.34	
	Shulv1	90.27 ± 0.17	9.77 ± 0.06	36.04 ± 0.14	3.03 ± 0.75	16.99 ± 0.1	39.58 ± 0.14	33.64 ± 0.03	
	Pushu53	88.61 ± 0.02	9.39 ± 0	31.36 ± 0.2	3.25 ± 0.08	13.74 ± 0.14	38.48 ± 0.13	42.19 ± 0.19	
	Ningcai	88.14 ± 0.4	10.66 ± 0.05	31.14 ± 0.08	2.49 ± 0.56	14.88 ± 0.02	38.42 ± 0.14	41.11 ± 0.45	
Peel **	Primary	22.4–22.3	4.74–4.76	6.40–6.49	2.33–2.65	9.47–9.70	55.2–56.1	76.4–77.0	[18]
	Blanched	14.2–14.0	3.65–4.01	8.11–8.20	1.28–1.35	6.45–6.60	29.6–30.1	79.9–80.4	
Residue of tuber after starch extraction	Beijing553	-	-	5.97 ± 0.43	0.45 ± 0.05	2.14 ± 0.01	23.81 ± 0.14	42.44 ± 0.04	[19]
	Jishu21	-	-	4.23 ± 0.05	0.52 ± 0.03	2.65 ± 0.08	17.15 ± 0.05	60.89 ± 0.11	
	Jishu71	-	-	4.05 ± 0.10	0.37 ± 0.06	1.59 ± 0.02	17.83 ± 0.08	59.41 ± 0.12	
	Jishu82	-	-	5.06 ± 0.05	0.38 ± 0.04	2.67 ± 0.03	24.49 ± 0.07	49.73 ± 0.05	
	Jishu98	-	-	4.37 ± 0.15	0.25 ± 0.03	2.09 ± 0.05	20.05 ± 0.06	53.76 ± 0.25	
	Jishu99	-	-	4.20 ± 0.49	0.21 ± 0.03	1.88 ± 0.04	16.33 ± 0.11	59.10 ± 0.06	
	Lvya18	-	-	3.38 ± 0.42	0.59 ± 0.02	1.99 ± 0.01	18.75 ± 0.04	53.53 ± 0.06	
	Weiduoli	-	-	6.11 ± 0.42	0.33 ± 0.02	3.02 ± 0.03	26.55 ± 0.04	43.45 ± 0.09	
	Xinong431	-	-	4.12 ± 0.16	0.37 ± 0.01	2.63 ± 0.15	23.35 ± 0.13	45.13 ± 0.11	
	Xu55-2	-	-	3.97 ± 0.01	0.48 ± 0.05	1.95 ± 0.04	25.82 ± 0.20	52.32 ± 0.06	

* The content of moisture is displayed as g/100 g fresh weight, while the contents of crude fibre, crude protein, crude fat, ash, dietary fibre, and carbohydrate are g/100 g dry weight; crude fibre was determined by ISO method 5498:1981 mentioned in Ref. [16]; carbohydrate content was calculated by subtracting the sum of crude fat, crude fibre, crude protein, and ash contents from 100; ** All data of the sweetpotato peel is displayed as the proportion of fresh weight (%); - No measurement has been performed in the corresponding reference.

**Table 2:** Chemical and monosaccharide composition (%) of dietary fibres derived from SP residues after starch isolation (modified and adapted from Ref. [19]) *.

Variety	Pectin	Hemicellulose	Lignin	Cellulose	Monosaccharide						
					Rhamnose	Arabinose	Galactose	Glucose	Xylose	Mannose	Uronic Acid
Beijing553	10.56 ± 0.18 g	12.09 ± 0.11 c	16.57 ± 0.06 f	27.66 ± 0.08 i	1.86 ± 0.05 f	4.29 ± 0.03 a	13.86 ± 0.28 a	57.29 ± 0.78 cd	2.63 ± 0.07 e	1.21 ± 0.06 de	18.86 ± 0.32 d
Jishu21	15.36 ± 0.11 ef	9.13 ± 0.04 g	22.61 ± 0.03 a	33.07 ± 0.30 b	2.39 ± 0.02 b	3.89 ± 0.09 bc	7.50 ± 0.09 f	58.95 ± 0.66 c	3.11 ± 0.04 cd	1.51 ± 0.05 c	22.65 ± 0.16 c
Jishu71	9.31 ± 0.61 h	12.01 ± 0.03 c	14.83 ± 0.05 i	36.54 ± 0.03 a	1.38 ± 0.01 g	3.96 ± 0.07 b	9.47 ± 0.22 d	65.73 ± 0.52 a	2.49 ± 0.06 e	2.14 ± 0.08 a	14.83 ± 0.23 e
Jishu82	21.02 ± 0.06 b	10.94 ± 0.10 e	8.96 ± 0.11 k	30.12 ± 0.13 f	2.24 ± 0.06 bc	3.79 ± 0.06 cd	8.52 ± 0.13 e	51.44 ± 0.41 f	2.92 ± 0.06 d	1.10 ± 0.08 e	26.35 ± 0.30 b
Jishu98	15.67 ± 0.30 e	9.09 ± 0.02 g	21.12 ± 0.11 c	27.90 ± 0.05 h	2.21 ± 0.05 cd	3.64 ± 0.05 e	8.75 ± 0.11 de	53.60 ± 0.37 ef	3.32 ± 0.08 c	2.08 ± 0.15 a	26.38 ± 0.11 b
Jishu99	9.01 ± 0.11 h	12.13 ± 0.05 c	17.41 ± 0.21 d	29.89 ± 0.11 g	1.51 ± 0.07 g	3.61 ± 0.05 e	12.11 ± 0.14 b	63.56 ± 0.43 b	3.17 ± 0.05 c	0.82 ± 0.03 f	15.22 ± 0.15 e
Lvya18	19.25 ± 0.17 c	13.32 ± 0.30 b	15.20 ± 0.06 h	31.93 ± 0.08 d	2.13 ± 0.04 de	3.88 ± 0.07 bc	10.91 ± 0.17 c	52.61 ± 0.29 f	3.68 ± 0.11 b	1.88 ± 0.07 b	25.91 ± 0.26 b
Weiduoli	18.29 ± 0.16 d	10.45 ± 0.05 f	22.01 ± 0.02 b	32.49 ± 0.04 c	2.30 ± 0.06 bc	2.90 ± 0.10 g	10.62 ± 0.18 c	54.47 ± 0.33 def	3.85 ± 0.09 ab	0.56 ± 0.04 g	25.31 ± 0.31 b
Xinong431	22.93 ± 0.16 a	8.70 ± 0.02 h	15.80 ± 0.07 g	25.92 ± 0.06 j	2.51 ± 0.08 a	3.19 ± 0.04 f	7.99 ± 0.17 ef	46.63 ± 0.51 g	4.07 ± 0.03 a	0.92 ± 0.05 f	34.69 ± 0.27 a
Xu55-2	15.13 ± 0.07 f	15.98 ± 0.07 a	13.94 ± 0.07 j	36.34 ± 0.21 a	2.01 ± 0.08 e	3.69 ± 0.11 de	14.21 ± 0.34 a	56.24 ± 0.62 cde	4.06 ± 0.08 a	0.47 ± 0.02 g	19.32 ± 0.19 d
Average	15.65 ± 0.06 e	11.38 ± 0.05 d	16.85 ± 0.13 e	31.19 ± 0.08 e	2.05 ± 0.05 e	3.68 ± 0.06 de	10.39 ± 0.27 c	56.05 ± 0.49 cde	3.33 ± 0.10 c	1.27 ± 0.09 d	22.95 ± 0.28 c

* Data is displayed as mean ± SD values, and different letters indicate the differences of data within the same column are statistically significant according to Duncan’s multiple-range test (*p* < 0.05).

## Data Availability

The original contributions presented in the study are included in the article, further inquiries can be directed to the corresponding author.
